# All and only CpG containing sequences are enriched in promoters abundantly bound by RNA polymerase II in multiple tissues

**DOI:** 10.1186/1471-2164-9-67

**Published:** 2008-02-05

**Authors:** Julian M Rozenberg, Andrey Shlyakhtenko, Kimberly Glass, Vikas Rishi, Maxim V Myakishev, Peter C FitzGerald, Charles Vinson

**Affiliations:** 1 Laboratory of Metabolism, National Cancer Institute, Bethesda, MD 20892 USA; 2 Physics Department, University of Maryland, College Park, MD 20742, USA; 3 Department of Dermatology University of Rochester School of Medicine, Rochester, NY 14642, USA; 4 Genome Analysis Unit, National Cancer Institute, Bethesda, MD 20892 USA

## Abstract

**Background:**

The promoters of housekeeping genes are well-bound by RNA polymerase II (RNAP) in different tissues. Although the promoters of these genes are known to contain CpG islands, the specific DNA sequences that are associated with high RNAP binding to housekeeping promoters has not been described.

**Results:**

ChIP-chip experiments from three mouse tissues, liver, heart ventricles, and primary keratinocytes, indicate that 94% of promoters have similar RNAP binding, ranging from well-bound to poorly-bound in all tissues. Using all 8-base pair long sequences as a test set, we have identified the DNA sequences that are enriched in promoters of housekeeping genes, focusing on those DNA sequences which are preferentially localized in the proximal promoter. We observe a bimodal distribution. Virtually all sequences enriched in promoters with high RNAP binding values contain a CpG dinucleotide. These results suggest that only transcription factor binding sites (TFBS) that contain the CpG dinucleotide are involved in RNAP binding to housekeeping promoters while TFBS that do not contain a CpG are involved in regulated promoter activity. Abundant 8-mers that are preferentially localized in the proximal promoters and exhibit the best enrichment in RNAP bound promoters are all variants of six known CpG-containing TFBS: ETS, NRF-1, BoxA, SP1, CRE, and E-Box. The frequency of these six DNA motifs can predict housekeeping promoters as accurately as the presence of a CpG island, suggesting that they are the structural elements critical for CpG island function. Experimental EMSA results demonstrate that methylation of the CpG in the ETS, NRF-1, and SP1 motifs prevent DNA binding in nuclear extracts in both keratinocytes and liver.

**Conclusion:**

In general, TFBS that do not contain a CpG are involved in regulated gene expression while TFBS that contain a CpG are involved in constitutive gene expression with some CpG containing sequences also involved in inducible and tissue specific gene regulation. These TFBS are not bound when the CpG is methylated. Unmethylated CpG dinucleotides in the TFBS in CpG islands allow the transcription factors to find their binding sites which occur only in promoters, in turn localizing RNAP to promoters.

## Background

The promoter region of genes is typically divided into two regions: the core or basal promoter region and the proximal promoter. The core promoter region stretches from around -50 bp to +20 bp and is the location in the promoter where the pre-initiation complex forms and the general transcriptional machinery assembles, including RNA polymerase II (RNAP). The proximal promoter extends from -200 bp to the transcriptional start site (TSS) and contains transcription factor binding sites (TFBS) that are critical for the recruitment of RNA polymerase II (RNAP) to DNA [2-4]. In mammalian genomes, the CpG dinucleotide occurs at 20% of the expected frequency [5] and is typically methylated both in cell cuture and animal tissues [6,7]. The exception is in CpG islands. CpG islands are defined as regions in the DNA at least 200 bp long where C+G comprise more than 50% of the nucleotides and CpG dinucleotides occur at greater than 60% the expected frequency (this represents roughly 8 or more CpGs in 200 bp) [8]. The presence of CpG islands is associated with gene regulatory regions [9] and in the promoters of genes generally correlates with binding by RNA polymerase II (RNAP) [9]. Promoters of housekeeping genes are constitutively bound by RNAP in all tissues while regulated promoters, either tissue specific or inducible, are selectively bound by RNAP in only certain tissue(s) or contexts respectively [2].

Three advances allow us to interrogate the genome-wide properties of promoters. First is the availability of complete genomic sequences. Second is the determination of full-length cDNAs that can identify the TSS and proximal promoter [10]. Third is the determination of the chromatin architecture of the genome by the identification of hypersensitive sites [11,12] or the location of particular proteins or their modified forms using chromatin immunoprecipitation followed by microarray analysis (ChIP-chip) [13]. Although ChIP-chip experiments have identified the location of RNAP and components of the preinitiation complex in particular tissues [9,14], these experiments have not been done systematically over a range of tissues.

We show that all and only CpG containing DNA sequences are associated with RNAP binding to the same promoter in multiple tissues. Many DNA sequences are more abundant near the TSS than elsewhere [15-18] and the six most abundant CpG containing sequences that are localized in proximal promoters are known TFBS and can predict RNAP binding to housekeeping promoters with similar accuracy as the presence of CpG islands.

## Results and discussion

### Binding of RNAP and H3K9me2 to mouse promoters in keratinocytes, liver, and heart ventricles

**Figure 1:**
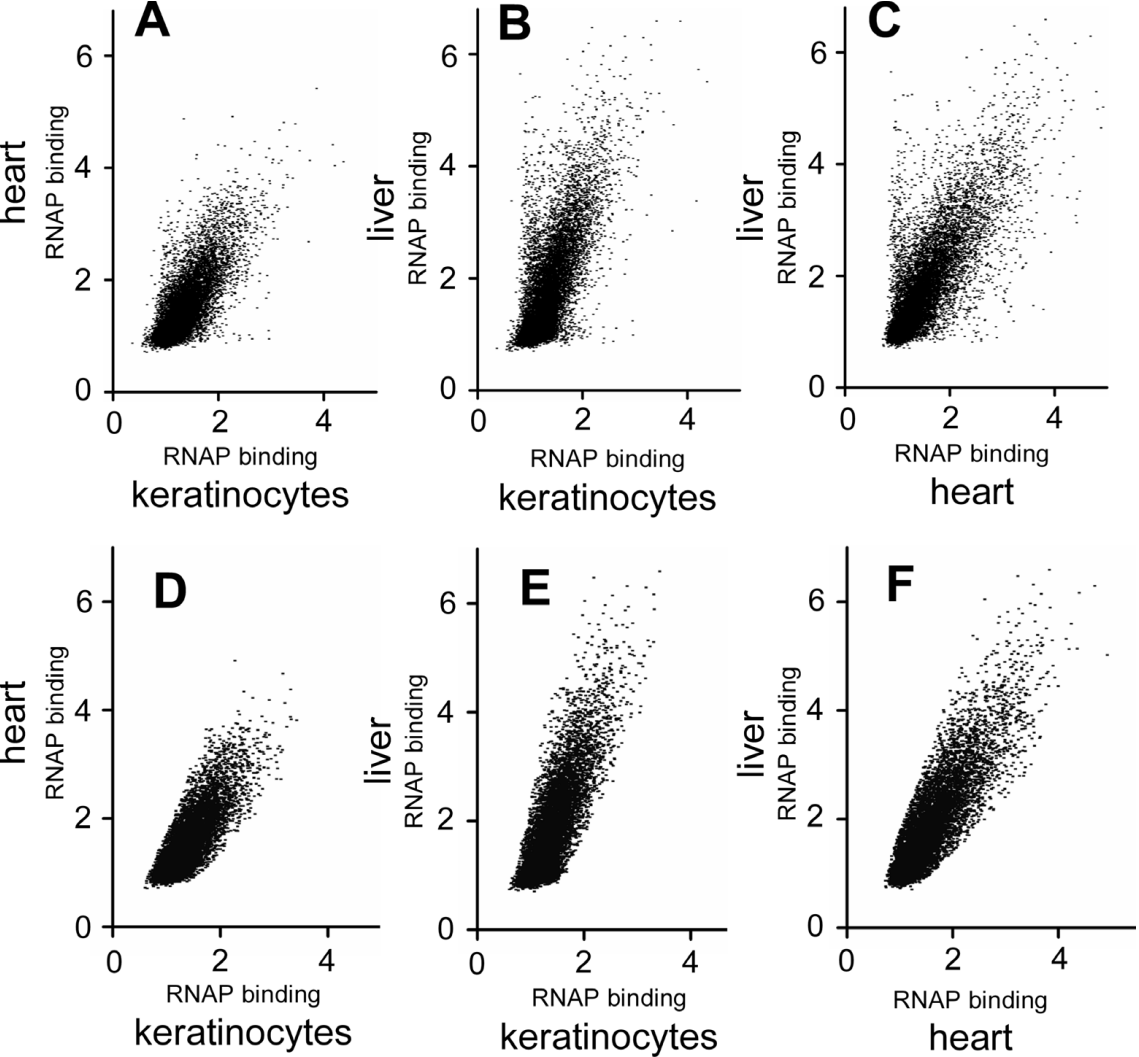
**A-C) RNAPbinding to 14,790 promoters from ChIP-chip data in different mouse tissues with each spot representing a single promoter**. **A) **keratinocytes versus heart ventricles (R = +0.76). **B) **keratinocytes versus liver (R = +0.73). **C) **heart ventricle versus liver (R = +0.76). **D-F) **RNAP binding to the 13,861 promoters with similar RNAP binding values in heart, liver and keratinocytes.

To gain insight into the DNA sequence properties of housekeeping promoters, we analyzed RNAP binding to promoters in three mouse tissues: primary skin keratinocytes, liver, and heart ventricles. Using ChIP-chip experiments [19], we determined the genomic localization of initiating (hypo-phosphorylated) RNAP [20,21] in all three tissues (Figure 1A–C). DNA from the RNAP ChIP analysis was amplified and hybridized to Nimblegen mouse promoter microarrays containing 15 probes spanning from -1,000 bp to +500 bp (see methods). Signal intensities were averaged for each promoter to produce a number representing binding at each promoter. This produced a graded binding of RNAP to promoter regions as has been previously observed [9,14,22]. Raw data for these ChIP-chip experiments can be found at the Vinson laboratory Web site [1]. We limited the following analysis of DNA sequence properties to the set of 14,790 promoters that contains neither similar/duplicated sequences nor a poorly annotated transcriptional start site (TSS).

To identify promoters that had similar RNAP binding values in all three tissues, we excluded genes where RNAP binding between any pair of tissues was significantly different. This excluded 534 tissue-specific (356 in liver, 131 in heart, and 47 in keratinocytes) promoters, and 395 with high RNAP binding in two of the three tissues. The remaining 13,861 promoters (94%) have similar RNAP binding in all three tissues, some being well bound by RNAP and others having little RNAP at the promoter (Figure 1D–F). For each of these 13,861 promoters, termed common RNAP promoters, RNAP binding values from the three tissues were normalized and averaged, producing a single number representing RNAP binding to a promoter across the three tissues.

To investigate the DNA sequence properties of the 13,861 common promoters (-1,000 bp to +500 bp) and determine potential transcription factor binding sites (TFBS) that are responsible for RNAP binding we analyzed the occurrences of 8 bp-long DNA sequences (8-mers) in common RNAP promoters. 8-mers were chosen because their length is similar to that of known TFBS. 8-mers were counted on the sense and anti-sense strands because, with the exception of TATA [23], 8-mers are not restricted to a single strand. Of all 32,896 8-mers (38% contain CpG) we extensively characterized the 12,208 most abundant 8-mers (see materials and methods) of which only 20% contained a CpG highlighting that the CpG dinucleotide is underrepresented even in promoter regions [15].

### All 8-mers enriched in promoters well bound by RNAP in multiple tissues contain a CpG dinucleotide

**Figure 2:**
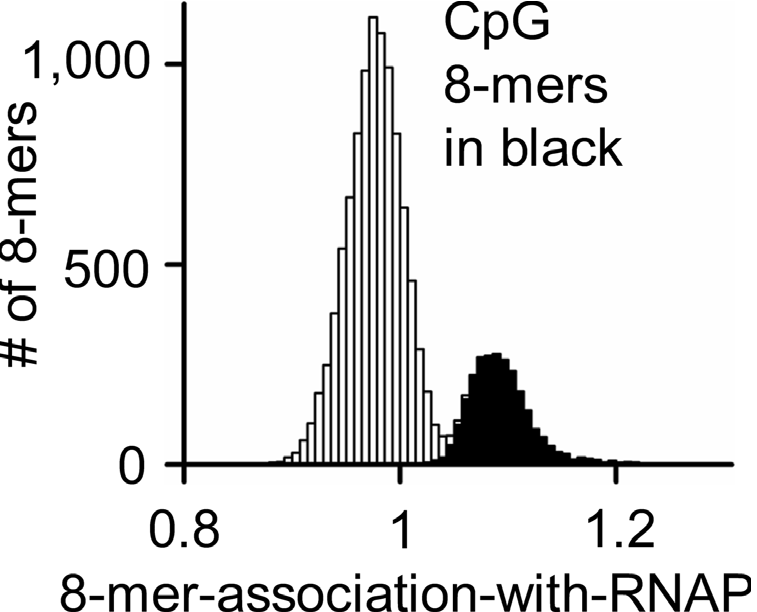
8-mer-association-with-RNAP for abundant 8-mers calculated for 13, 861 common promoters between -1,000 bp and +500 bp. 8-mers that contain a CpG are noted in black.

To measure 8-mer enrichment in promoters commonly bound by RNAP, we calculated the term "8-mer-association-with-RNAP" for all 8-mers. This term is the average RNAP binding to promoters that contain a particular 8-mer normalized by the average RNAP binding to all common promoters. The value "8-mer-association-with-RNAP" is calculated for each 8-mer by first identifying all the promoters that contain that particular 8-mer, and then averaging the RNAP binding values of those promoters. These values are then normalized by dividing by the average of the RNAP binding values of all common promoters. A histogram of these values has a bimodal distribution. 20% of 8-mers are associated with high RNAP binding to common RNAP promoters (Figure 2). This result suggests that the graded binding of RNAP to promoters is caused by a combination of 8-mers, some of which favor RNAP binding and others which do not. The region of the promoter (-1,000 bp to +500 bp) critical for the observed bimodal distribution extends from -600 bp to +400 bp (see Additional file 1). Strikingly, nearly all the 8-mers that are associated with RNAP binding contain the CpG dinucleotide while virtually none of the remaining 8-mers contain a CpG. In contrast to the CpG dinucleotide, the other dinucleotides did not exclusively occur in either part of the bimodal distribution (Additional file 2). A spreadsheet containing the 8-mer-association-with-RNAP for all 8-mers is included in the supplementary material (Additional file 5).

**Figure 3:**
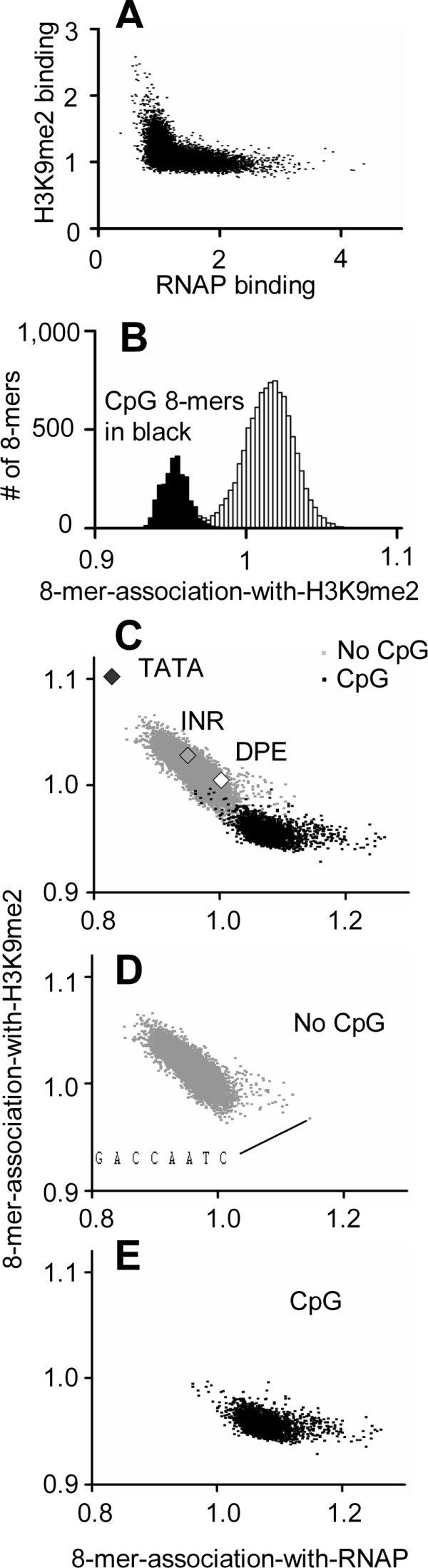
**A) Binding ofRNAPvs. H3K9me2 (R = -0.50) in mouse tissue culture keratinocytes.B) **8-mer-association-with-H3K9me2 for 12,208 abundant 8-mers, calculated for 14,790 promoters between -1,000 bp and +500 bp; CpG containing 8-mers are noted in black. **C-E) **8-mer-association-with-RNAP vs. 8-mer-association-with-H3K9me2. **C) **All 8-mers. The association-with-RNAP and the association-with-H3K9me2 for the core promoter elements at their unique position in promoters is presented for TATA (TATAWAAR), INR (YYANWYY) and DPE (RGWYV). **D) **8-mers without a CpG. **E) **8-mers with a CpG.

To evaluate if other types of promoters have a different enrichment of 8-mers, we examined the transcriptionally inactive genes marked by a post-translationally modified form of histone 3, H3K9me2 (lysine 9 containing a dimethyl group) [24,25]. In keratinocytes, ChIP-chip identification of H3K9me2 genomic localization negatively correlated with RNAP (correlation coefficient, R = -0.50) (Figure 3A). The 8-mer-association-with-H3K9me2 also had a bimodal distribution with the CpG containing 8-mers associating the least with H3K9me2 binding (Figure 3B). As anticipated (comparing Figure 2 and 3B), practically all the 8-mers most associated with common RNAP binding also are least associated with H3K9me2 binding (Figure 3C). Similar results were obtained when all 8-mers were examined (Additional file 3A–E).

The 8-mers with and without a CpG were plotted separately to highlight the few 8-mers that are the exception to the general conclusion that only CpG containing sequences are associated with RNAP binding to a promoters (Figure 3D–E). The most notable exception is the GACCAATC 8-mer, a CCAAT sequence that is enriched in housekeeping promoters.

Previous work indicated that ~50% of human promoters bound by RNAP contain the INR and DPE consensus sequences between -200 bp and +200 bp [9]. To see if these non-CpG-containing sequences were also exceptions to our general conclusion, we calculated the association-with-RNAP and association-with-H3K9me2 for TATA, INR and DPE in the set of promoters with similar RNAP binding values in the three tissues we have examined. This was accomplished by averaging the binding values of those promoters that contained the consensus sequence at the expected position [3]. In mouse, the consensus TATA is uniquely positioned in only 1.8% of promoters and has a very high association-with-H3K9me2 binding to promoters. The INR was uniquely positioned in only 9% of promoters and is associated with H3K9me2 bound promoters. DPE is not uniquely positioned in promoters, but occurs in 19% of promoters at the expected location and is also associated with H3K9me2 binding (Figure 3C). This suggests that TATA, INR and the DPE are not important for RNAP binding to promoters in multiple tissues. Presumably these sequences are important for tissue-specific gene expression.

### CpG sequences are also associated with mRNA expression

**Figure 4:**
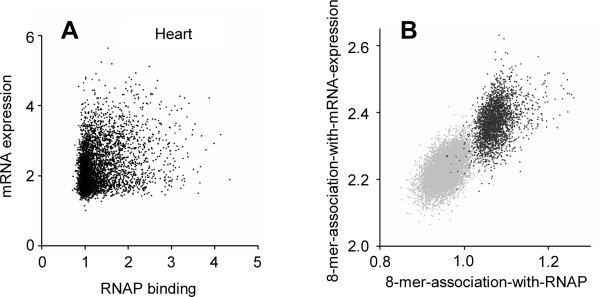
**A) RNAPbinding to promoters vs. mRNA expression for 4,522 promoters with common identifiers.B) **8-mer-association-with-RNAP vs. 8-mer-association-with-mRNA-expression for abundant 8-mers calculated using the 4,522 promoters graphed in (A). CpG-containing 8-mers are notated in black.

We examined whether RNAP binding to the promoter correlates with mRNA expression levels in the genes whose promoters are bound similarly by RNAP in the three tissues examined. mRNA expression data for heart ventricle was obtained [26] and compared to RNAP binding levels for the 4,522 promoters that share a common identifier (Figure 4A). We calculated the 8-mers-association-with-mRNA-expression and found the same 8-mers associated with RNAP binding to promoters also associated with mRNA expression (Figure 4B). Thus, CpG-containing 8-mers are most enriched in promoters that have the highest RNAP binding and mRNA expression.

### Sequences most enriched in tissue-specific promoters do not contain a CpG

**Figure 5:**
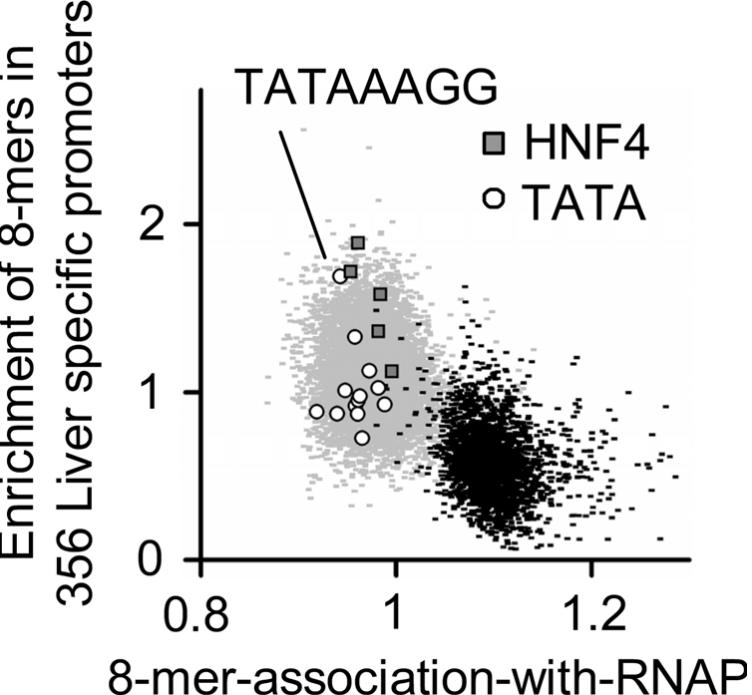
**8-mer-association-with-RNAPvs. 8-mer enrichment in 356 liver specific promoters for abundant 8-mers.** Highlighted 8-mers contain TATA sequences (STable 1 in Additional file 4) and the liver specific HNF4 binding sites (8-mers containing TGACCT). The CpG containing 8-mers are plotted in black.

The DNA sequence properties of tissue specific promoters that were well bound by RNAP in only one tissue were compared with housekeeping promoters well bound by RNAP in all three tissues. The abundant 8-mers most enriched in the 356 liver specific promoters do not contain CpG and were different than those associated with RNAP binding in all three tissues (Figure 5, Additional file 3F). As expected, the liver-specific transcription factor HNF4 is enriched in the liver-specific genes. The fact that TATA sequences are also enriched in the liver specific genes is consistent with suggestions that it is a marker for tissue specific, not constitutive gene expression [15,27]. Some CpG containing 8-mers are enriched in the liver specific genes indicating that in addition to their housekeeping function, these sequences also mediate tissues specific gene expression. This has been well documented for the CRE (TGACGTCA) [28,29].

### Non-random distribution of 8-mers in promoters

**Figure 6:**
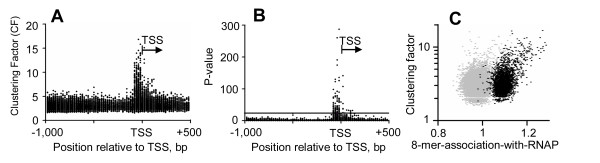
**A) A measure of non-random distribution termed a Clustering Factor (CF) is plotted in the most populated bin for 8-mers with at least 20 members in the most populated 20 bp bin (abundant 8-mers).** Note the dots between -100 bp and the TSS with large CF values representing 8-mers that are more abundant near the TSS than elsewhere. **B) **A probability term P for the 8-mers in (A). A P value of 24 means that the distribution of the 8-mer has a less than 10^-24 ^chance of being random. **C) **Non-random distribution of 8-mers (Clustering Factor) vs. 8-mer-association-with-RNAP for abundant 8-mers.

If the 8-mers that associate with RNAP binding are TFBS, they may be localized in the proximal promoter as has been observed in human [15,16] and Drosophila promoters [23]. We thus determined the "Clustering Factor" (CF, a measure of non-random distribution between -1,000 bp and +500 bp) [15,23] for abundant 8-mers in promoters, and compared it to 8-mer-association-with-RNAP. Some 8-mers were preferentially localized near the TSS (Figure 6A–B). The 8-mers most associated with promoters commonly bound by RNAP had a high CF (Figure 6C, Additional file 3G). However, there was also a class of 8-mers with high CFs but low 8-mer-association-with-RNAP values that may represent TFBS involved in regulated gene expression.

The 120 8-mers with the statistically highest CF (Figure 6B) that localize upstream of the TSS could be manually grouped into ten consensus motifs that are known TFBS: ETS, NRF-1, E-Box, BoxA, CRE, SP1, KLF, CCAAT, TATA, and CRE-T (STable 1 in Additional file 4), six of which contain a CpG dinucleotide (ETS, NRF-1, E-Box, BoxA, CRE, and SP1). A similar analysis has identified that these ten motifs localize to the proximal promoter in human promoters [15]. The six motifs that contain a CpG in the consensus motif (ETS, NRF-1, E-Box, BoxA, CRE, and SP1) always positively correlated with each other in the proximal promoter, exceeding expectations by up to two fold (STable 2A in Additional file 4), were enriched in the 20% of promoters best bound by RNAP in all three tissues (STable 2B in Additional file 4), and were underrepresented in H3K9me2 marked promoters (STable 2C in Additional file 4). ETS, NRF-1, and BoxA correlate the best with RNAP binding to promoters in multiple tissues (STable 2B in Additional file 4). Of the ten identified motifs, only TATA and CRE-T were enriched in the 20% of promoters best marked by H3K9me2 in keratinocytes (STable 2C in Additional file 4). To see if these TFBS play some specific role in mRNA expression or RNAP binding, we calculated the association-with-mRNA-expression and association-with-RNAP for the consensus sequences of these TFBS (Table 1). As expected, the CpG-containing TFBS have high association values for both mRNA expression and RNAP binding.

**Table 1:** Association of the 10 localized motifs with RNAP binding and mRNA expression.

**Motif**	**Sequence**	**8-mer-association-with-RNAP**	**8-mer-association-with-mRNA expression**
BoxA	TCTCGCGA	1.30	2.50
NRF-1	GCGVTGCG	1.24	2.44
ETS	VCCGGAARY	1.21	2.39
CRE	TGACGTCA	1.19	2.32
SP-1	CCCCGCCC	1.14	2.38
E-Box	YCACGTGA	1.10	2.28
CCAAT	RRCCAATSR	1.04	2.27
KLF	CCCCTCCC	1.04	2.28
TATA	TATAAAD	0.96	2.22
CRE-T	TGATGTCA	0.90	2.17

Column one contains the name of the motif; column two contains the DNA sequence of the motif; column three is the 8-mer-association-with-RNAP for promoters (-1,000 bp to +500 bp) commonly bound by RNAP in the three tissues examined ranked in order by association; column four is 8-mer-association-with-mRNA-expression.

### CpG islands can be defined by two or more of the six CpG containing TFBS

**Figure 7:**
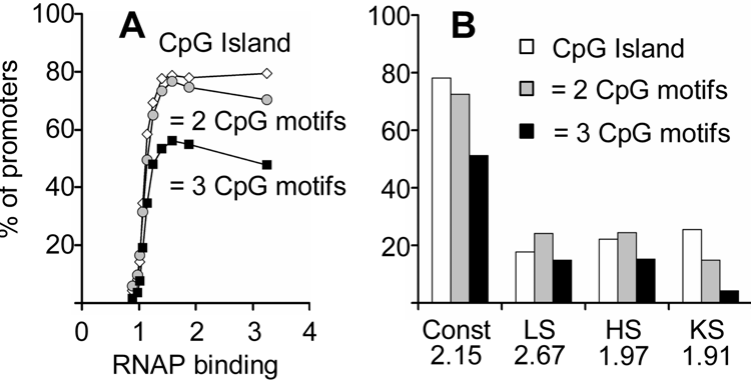
**A) Fraction of promoters that contain particular sequences between -200 bp and TSS: 1) CpG island, 2) two or more of six CpG containing motifs (SP1: CCCGCC, CCGCCC, CGCCCC; ETS: CCGGAA, GCGGAA; NRF-1:CGCATGCG, CGCGTGCG, CGCCTGCG; BoxA: TCTCGCG, CTCGCGA; CRE: ACGTCA; E-Box: CACGTG), 3) three or more of the six motifs.B) **Fraction of promoters that contain particular motifs: top 20% of common RNAP promoters (Const), liver specific (LS), heart ventricle specific (HS), and keratinocyte specific (KS) promoters. Average RNAP binding for each class is presented.

Previous work has suggested that housekeeping genes can be defined by the presence of a CpG island in the promoter region [8], but the DNA sequences properties of CpG islands has not been described. We evaluated if the presence of the six CpG consensus motifs in proximal promoters (-200 bp to the TSS) predicts RNAP binding to promoters commonly bound by RNAP and compared these results with the occurrence of a CpG island between -200 bp to the TSS (Figure 7A). The results demonstrate that the presence of any two of these motifs recapitulates the discrimination based on the presence of a CpG island in regards to RNAP binding to common promoters. In order to compare these two measures, we grouped promoters into ten equal size groups with increased RNAP binding. 80% of promoters in the group best bound by RNAP contain a CpG island and a similar number contain two or more of the six motifs (Figure 7A). Similarly, only 5% of promoters with the lowest RNAP binding values are CpG islands, and only about 5% have two or more motifs (Figure 7A). The presence of three or more of these motifs produced a lower positive hit rate in the best bound group (48%) but occurred in only 1% of promoters not bound by RNAP. Therefore, our analysis suggests that CpG islands have predictive value in defining housekeeping genes because of the presence of these six TFBS motifs. These six motifs do not account for all CpGs in CpG islands. Some of the other CpGs are known TFBS but the function of the rest remains unclear. They could be sequences that persist because they are protected from methylation and ultimate destruction or they could be involved in the higher-level regulatory processes that have been proposed for CpG islands [30]. In contrast to promoters well bound by RNAP in multiple tissues, only 20% of tissue specific proximal promoters are CpG islands and similarly only 20% contain two or more of these six motifs. This indicates that these six motifs correlate with promoters that are bound by RNAP in multiple tissues and not tissue specific promoters (Figure 7B).

### Nuclear extracts do not bind TFBS with a methylated CpG

Methylation of CpG dinucleotides in CpG islands inhibits promoter activity and occurs in many cancers where the oncogenic event is the transcriptional suppression of tumor suppressor genes [30]. One simple explanation is that CpG methylation inhibits TFs from binding their TFBS resulting in promoter inactivity. A more prevalent, but not mutually exclusive view suggests that a more active mechanism is functioning in which methyl binding proteins bind methylated CpGs to facilitate chromatin mediated occlusion of the promoter [30,31]. The effect of CpG methylation on the function of five of the six CpG containing TFBS (DNA binding and/or transcriptional potential) that localize in the proximal promoter has been described. The one exception is BoxA, for which the effect of CpG methylation on DNA binding has not been reported in the literature. In general, methylation inhibits the activity of CpG containing TFBS [32]. CpG methylation is reported to inhibit the function of a CRE [33], ETS [34], NRF-1 [35], and E-Box [36]. Other CpG containing motifs are also inhibited via methylation including AP2 [37] and CTCF [38]. Methylation of the CpG in the SP1 motif, the most abundant CpG containing motif, is reported to either not affect DNA binding [39-41], affect binding when a cytosine flanking the CpG is methylated [41,42] or inhibit binding [43].

**Figure 8:**
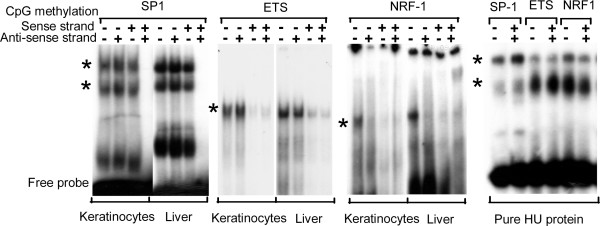
EMSA using keratinocyte and liver nuclear extracts and pure HU protein with 28 bp double stranded oligonucleotides containing on the sense strand a canonical SP1 (GGGGCGGG), ETS (CCGGAA), and NRF-1 (GCGVTGCG) site where the cytosine in the CpG is non methylated (-/-), hemi-methylated (-/+), hemi-methylated (+/-), or methylated (+/+).

We observe that CpG methylation of a canonical SP1, ETS, or NRF-1 site abolishes DNA binding of nuclear extracts isolated from either liver or primary keratinocytes (Figure 8). When both DNA strands of a canonical SP1 site are methylated, nuclear extract binding are abolished. For ETS, methylation of a one strand of DNA is sufficient to abolish DNA binding while for NRF-1, methyation of both CpGs in the canonical site on either strand is sufficient to abolished binding. As a control, we show that the methylated SP1 oligonucleotides could bind to the non-specific prokaryotic protein HU. Reexamination of previous reports indicates that SP1 methylation causes a modest decrease in SP1 binding that our experimental system is able to demonstrate more dramatically.

## Conclusion

We identified promoters that are bound similarly by RNAP in multiple tissues and determined the association between the presence of 8-mers in these promoters and the extent of RNAP binding to the promoter. Looking at RNAP binding to housekeeping promoters, we observed a bimodal distribution: only 8-mers with the CpG dinucleotide are in the class of sequences most associated with RNAP binding and only 8-mers without a CpG are in the class least associated with RNAP binding. An implication of this observation is that knowing if a TFBS contains a CpG reveals aspects of its biological function. If the TFBS contains a CpG, it is involved in constitutive gene expression and if the TFBS does not contain a CpG, it is involved in regulated gene expression. This insight will help identify potential functions for transcription factors when their TFBS is identified. Additionally, if a transcription factor shows degeneracy in its TFBS [44,45], binding to a CpG sequence and a similar sequence without a CpG, it suggests that this transcription factor is involved in both constitutive and regulated gene expression. This is observed for the CRE and CRE-T sequences, two sequences that are localized in the proximal promoter and differ by a single base: CRE contains a CpG (TGACGTCA) while CRE-T does not (TGA**T**GTCA). The CREB protein binds both sequences well (data not shown) but the two sequences correlate very differently with RNAP binding suggesting that the CREB transcription factor can regulate either constitutive gene expression by binding the CRE sequence or regulated gene expression by binding the CRE-T sequence.

In vertebrates CpG dinucleotides are rare and usually are methylated on the cytosine but do occur at close to the expected frequency in clusters called CpG islands where the CpGs remain unmethylated [30,46]. These CpG islands often occur in promoters of housekeeping genes [8,9]. We show that the presence of two or more of any of the six CpG containing TFBS (SP1, ETS, NRF-1, CRE, E-Box, and BoxA) in the proximal promoter can predict RNAP binding to housekeeping promoters as accurately as the presence of a CpG island in the proximal promoter.

Methylation of the CpG in the TFBS has been found to inhibit the DNA binding for five of the six TFBS that are abundant and localize in proximal promoters suggesting this may be a general result for CpG containing TFBS. Methylation dependent inhibition of transcription factor binding to DNA has two implications. First, the transcription factors that are critical for the activation of housekeeping genes solve the problem of finding their TFBS in the genome by only binding to unmethylated TFBS. Since most CpGs in the genome are methylated, the only places these transcription factors can bind are in the unmethylated CpG islands in promoters. Second, the pathological methylation of CpG dinucleotides in CpG islands, as occurs in many cancers [30], would prevent these abundant transcription factors from binding their TFBS thus causing the promoters to become inactive. This could be a critical initial step that subsequently allows CpG methyl binding proteins to bind to methylated CpGs and actively repress a promoter [31].

## Methods

### Promoter annotation

Mouse (*Mus musculus*) annotation data and genomic DNA sequences for the region -1,000 bp to +500 bp, relative to the annotated transcription start site (TSS), were downloaded from the UCSC Genome Browser site (*version mm5, May 2004*). This dataset contains the putative promoter regions of 26,000 genes that are represented on the MM5 minimum promoter mouse Nimblegen ChiP-chip array. However, since the TSS for many of these genes is poorly annotated (e.g. the TSS is the same as the translation start), we refined this dataset to include only those genes where the distance between the TSS to the translation start (ATG) was greater than 30 nucleotides. This reduced the total number of putative promoter regions to 15,180. We further reduced this number by excluding promoter with gaps greater than 200 bps and the blastclust program was used to confirm that this dataset did not contain multiple copies of the same DNA sequences resulting in 14,790 promoters.

The 14,790 analyzed promoters are a biased subset of the 26,000 promoters on the ChIP-chip array. The annotated promoters are enriched 1.3 fold for the 20% of promoters best bound by RNAP and depleted by 2 fold for H3K9me2 bound promoters. This could reflect that the H3K9me2 genes are not universally expressed and full-length cDNA data does not exist for them, preventing identification of a TSS.

### Clustering Factor (CF) calculation

To determine if a DNA sequence has a non-random distribution (i.e. clustered), we used an automated method of detecting and quantifying peak height as described previously [15]. Abundant 8-mers contained 20 or more members in a 20 base pair window in the 14,970 examined promoters.

### Cultures of primary keratinocytes

Primary keratinocytes were isolated from newborn FVB mice epidermis [47]. Primary keratinocytes were seeded at a density of 0.6 pelt or 5 × 10^6 ^cells per 100-mm dish in Ca^+2 ^and Mg^+2 ^free EMEM (Cambrex Bio Science Walkersville, Inc), supplemented with 8% Chelex (Bio-Rad, Richmond, CA) treated FBS (Atlanta Biologicals, Inc), 0.2 mM Ca2+ and Antibiotic-antimycotic. After 20 h, cultures were washed with PBS and switched to the same medium containing 0.05 mM Ca^+2^. After three days cells were used for ChIP.

### Liver and heart samples

Tissues from 5 adult FVB mice were frozen and ground in fine powder in liquid nitrogen. After nitrogen evaporation, samples were moved into a 50 ml conical tube and 10 mls of 1% formaldehyde in PBS was added and samples incubated for 10 minutes at 37°C with vortexing. 125 mM glycine was added for 5 minutes, cells were washed in PBS with 1 mM PMSF once, dounced in Lyzis buffer (5 mM PIPES pH 8.0 85 mM KCL 0.5% NP40 1 mM NF 1 mM NaVa Roche protease inhibitors cocktail) and re-suspended in 200 *μ*l Nuclear lysis buffer (50 mM Tris-Cl pH 8.1 10 mM EDTA, 1% SDS proteases and phosphates inhibitors as above). DNA was sheared by sonication to yield fragments from 3,000 to 300 bp. Samples were centrifuged and supernatants were diluted 6 times (0.01% SDS, 1.1% Trition × 100, 1.2 mM EDTA, 16.7 mM Tris-Cl pH 8.1, 167 mM NaCl) and used for ChIP.

### Chromatin immunoprecipitation

Chromatin immunoprecipitation (ChIP) was performed using antibodies against RNAP from Covance, (8WG16) that recognizes the unphosphorylated form of RNAP, H3K9me2 from Upstate (07–441), and CREB using a mixture of antibodies from Santa Cruz (sc-186) and Upstate (06–863), c-Jun from Santa Cruz (sc-1694). The ChIP protocol was from P. Farnham [19,48]. For immunoprecipitation, we used protein G agarose beads (Invitrogen). Starting with 2 × 10^6 ^cells, we typically isolate 1 ng of ChIP DNA for RNAP and 5 ng for histone H3K9me2.

### ChIP DNA amplification and hybridization

Protocol for random DNA amplification [49,50] was adapted from DeRisi lab. We used primers conjugated with Cy3 or Cy5. After amplification 10–15 ug of DNA was purified using Quiagen PCR purification Kit, concentrated by isopropanol precipitation and dried for 5 min under vacuum. DNA was dissolved in 3 *μ*l water, mixed with Component A and Hybridization buffer (Nimblegen) according to manufacturer instructions. Amplified ChIP DNA was hybridized to Nimblegen MM5 min Mouse promoter microarrays containing 400,000 oligos interrogating 26,000 promoters. Arrays were washed in 45C 0.2%SDS, 0.2%SSC for 15 sec, in the same buffer at room temperature for 2 min, 0.2%SSC for one minute, 0.05% SSC for 15 sec. Arrays were dried by centrifugation and scanned using Axon 4000B scanner. Images were processed with NIMBLESCAN (Nimblegen) using default settings. Average of enrichment for fifteen spots representing one promoter were used as a measure of "binding" for a protein. We averaged binding of RNAP and H3K9me2 from two independent hybridizations for each tissue using independent biological samples. Correlation coefficients for keratinocytes replicates were: RNAP – 0.79, H3K9me2 – 0.67 and for RNAP ChIP's from liver samples: 0.86; heart samples: 0.83.

### Electrophoretic Mobility Shift Assay (EMSA)

Following PAGE purified 28 base pairs long oligonucleotides, the sense strand, with their complimentary strands were purchased from Sigma-genosys (USA).

SP-1: GTCAGTCAGGGGG(C/C^m^)GGGGCATCGGTCAG

ETS: GTCAGTCAGAC(C/C^m^)GGAAGTTATCGGTCAG

NRF-1: GTCAGTCAGA(C/C^m^)GCCTG(C/C^m^)GTATCGGTCAG

A single consensus binding site for each transcription factor containing either nonmethylated (C) or methylated cytosine (C^m^) (1 methyleted cytosine in SP-1 and ETS and 2 in NRF-1) is underlined. Sense strands of non-methylated and methylated oligos were end labeled with (*γ*^32^P) ATP (5000 mCi/mmol; MP Biomedical) using T4 PNK enzyme (New England Biolabs). Equimolar labeled sense and complimentary cold anti-sense oligos were annealed by heating the mixture in annealing buffer to 65°C for 15 minutes and snap cooling it on ice for 2 minutes followed by incubation at room temperature for 15 min. Annealing resulted in four types of labeled double stranded oligos (1 non-methylated, 2 hemi-methylated oligos and 1 methylated oligo) and these were used for EMSA.

Nuclear extract was prepared from mouse liver and cultured mouse primary keratinocytes [51]. In 20 *μ*l of reaction sample, 7 pg of labeled oligonucleotide (50,000 cpm) was added to 5 *μ*g of nuclear extract, and incubated in binding buffer (10 mM HEPES, 80 mM KCl, 0.05 mM EDTA, 6% glycerol, 1 mM DTT and 1 mM MgCl_2_) at 37°C for 20 min. Samples were separated on a 5% native PAGE gel in 0.25 × TBE at 150 V for 1.5 hrs. Gels were dried and exposed for autoradiography. For EMSA involving E. coli HU protein, a kind gift from Shankar Adhya, 30 nM of HPLC purified recombinant HU was incubated in binding buffer (25 mM Tris-HCl pH 8.0, 50 mM KCl, 0.5 mM EDTA, 2.5 mM DTT, 1 *μ*g BSA) with 7 pg of labeled double stranded oligo in a total volume of 20 *μ*l and complex was separated on 7.5% native page (0.25 × TBE, 150 V, 1.5 Hrs), dried and autoradiographed.

### 8-mer-association-with-binding

To find the "8-mer-association-with-binding" (*b*_8_), we averaged the binding values of the promoters (*b*_*p*_) whose sequence contained that 8-mer and divided by the average of the binding values to the promoters ($\bar{b_{p}}$).

$$b_{8}=\frac{\sum_{p}\partial_{8p}b_{p}}{\bar{b_{p}}\sum_{p}\partial_{8p}}$$

Where *p *is the promoter in question. ∂_8*p *_is equal to one if the 8-mer occurs in the promoter sequence and zero otherwise. Summing over *p *implies summing over all the promoters in the set in question.

### Promoters with similar RNAP binding

In order to identify promoters with similar RNAP binding in two tissues, we rotated the data so that the best-fit line was the 45-degree line through the origin. The two-dimensional rotation matrix is:

$$\left|\begin{array}{cc} \cos\theta & \sin\theta \\ -\sin\theta & \cos\theta \end{array}\right|$$

where *θ *is the angle by which we rotated the coordinates in the two-dimensional plane. For a given pair of data sets, this angle can be determined by subtracting the angle of the best-fit line from 45 degrees. For each data point, the rotated values are calculated by operating the rotation matrix on the original data point. The line can be forced to the origin by adding or subtracting the value of the vertical-intercept of the best-fit line from the vertical data before the rotation. The new "rotated binding values" are then determined by operating on the original binding values:

$$\left|\begin{array}{c} b_{A}^{rotated}\\ b_{B}^{rotated} \end{array}\right|=\left|\begin{array}{cc} \cos\theta & \sin\theta \\ -\sin\theta & \cos\theta \end{array}\right|\left|\begin{array}{c} b_{A}\\ b_{B} \end{array}\right|$$

In order to assure that the rotation was robust and not heavily influenced by outliers in the data set, we temporarily removed data more than one standard deviation from the original best fit line. If the best-fit line of the transposed data still maintained its 45-degree angle within some small error range, we concluded the data was successfully rotated. If not, then we repeated the procedure using the new rotated values and only those points within one standard deviation of the best-fit line to determine the new rotation angle and intercept adjustment. This was repeated until the best-fit line did not significantly alter with the removal of data points more than one standard deviation from 45 degree line.

In our case we had RNAP binding values for three distinct tissues: primary mouse keratinocytes, heart ventricle, and liver. We knew that the results are similar in all three tissues, with the exception of genes involved with tissue-specific expression in those tissues. We rotated the data by pairs in the method described above. This took several iterations since the rotation of one pair might affect the values of another pair. The end result was new "rotated binding values" for the promoters in each of the three tissues. These values were then averaged to produce the "Average RNAP binding" of that promoter in all three tissues.

### Determining Tissue Specific Promoters

Promoters which were more than two standard deviations off of the 45-degree best-fit line (as determined above) through any of the three pair of data (liver-heart, liver-keratinocytes, and heart-keratinocytes), were considered "tissue-specific" (not commonly bound). Of our original set of 14,790 promotes, 929 were not commonly bound by RNAP in all three tissues, leaving 13,861 promoters which were commonly bound in all three tissues. Of 929 promoters that were not commonly bound by RNAP, tissue specific promoters were selected based on following criteria using the raw RNAP binding values:

356 liver specific promoters: L > 1.5 × H, L > 1.5 × K, H< 1.5 (raw RNAP binding value), K < 1.5

131 heart specific promoters: H > 1.3 × L, H > 1.3 × K, L < 1.5, K < 1.5

47 keratinocytes specific promoters: K > 1.5 × L, K > 1.5 × H, H < 1.5, L < 1.5

Where L stands for RNAP binding value in liver, H is RNAP binding in heart and K – RNAP binding in keratinocytes.

## Authors' contributions

JMR did the ChIP-chip experiments and helped in data analysis, AS, KG, and PCF helped in data analysis, VR did the SP1 gel shift, MVM helped in ChIP-chip experiments, and all authors helped in manuscript preparation. All authors read and approved the final manuscript.

## Supplementary Material

Additional file 1 The region of the promoter critical for the bimodal distribution of the 8-mer-association-with-RNAP. Histogram of the 8-mer-association-with-RNAP between -1,000 bp and +500 bp and in 200 bp increments from -1,200 bp to +1,000 bp for abundant 8-mers in the common RNAP promoters. 8-mers that contain a CpG are noted in black. Click here for file

Additional file 2 Distribution of the 8-mer-association-with-RNAP for 8-mers containing particular dinucleotide. Histograms of the 8-mer-association-with-RNAP between -1,000 bp and +500 bp for abundant and all 8-mers with 8-mers containing each of the 10 dinucleotides noted in black. Click here for file

Additional file 5 8-mer-association-with-RNAP for all 8-mers. Spreadsheet containing the 8-mer-association-with-RNAP for all 8-mers. Click here for file

Additional file 3 Data presented at figures 2, 3(D–E), 5 and 6C for all 8-mers. The data presented at figures 2, 3(D–E), 5 and 6C is shown here for all 8-mers. Histograms and scatter plots for 8-mer-association-with-RNAP vs. 8-mer-association-with-H3K9me2, enrichment of 8-mers in 356 liver specific promoters vs. 8-mer-association-with-RNAP, clustering factor vs. 8-mer-association-with-RNAP. Click here for file

Additional file 4 Supplementary tables. Table 1 shows the 120 statistically most non-randomly distributed sequences placed into 10 groups. Table 2 shows co-occurrence of the 10 proximal promoter motifs between -200 bp and the TSS in 14,790 mouse promoters, top 20% of common RNAP promoters and top 20% of promoters best bound by H3K9me2. Click here for file
