# Supplementary material for: All and only CpG containing sequences are enriched in promoters abundantly bound by RNA polymerase II in multiple tissues
Source: BMC Genomics. 2008 Feb 5;9:67. doi: 10.1186/1471-2164-9-67 (PMC2267717; doi:10.1186/1471-2164-9-67)
Supplement: Additional file 1 — The region of the promoter critical for the bimodal distribution of the 8-mer-association-with-RNAP. Histogram of the 8-mer-association-with-RNAP between -1,000 bp and +500 bp and in 200 bp increments from -1,200 bp to +1,000 bp for abundant 8-mers in the common RNAP promoters. 8-mers that contain a CpG are noted in black. [file 1471-2164-9-67-S1.ppt]

## Slide 1
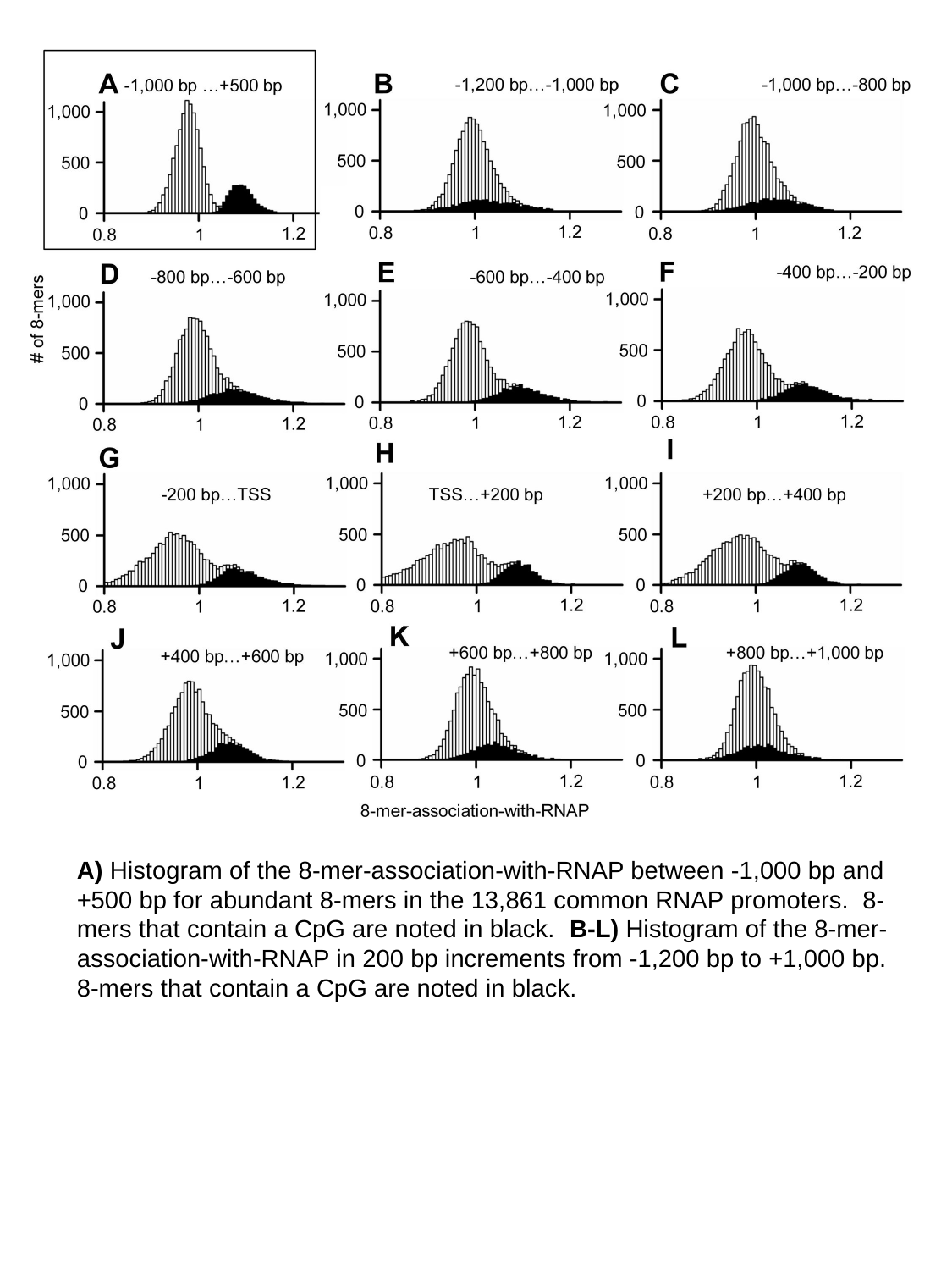

A) Histogram of the 8-mer-association-with-RNAP between -1,000 bp and +500 bp for abundant 8-mers in the 13,861 common RNAP promoters. 8-mers that contain a CpG are noted in black. B-L) Histogram of the 8-mer-association-with-RNAP in 200 bp increments from -1,200 bp to +1,000 bp. 8-mers that contain a CpG are noted in black.
