# Supplementary material for: All and only CpG containing sequences are enriched in promoters abundantly bound by RNA polymerase II in multiple tissues
Source: BMC Genomics. 2008 Feb 5;9:67. doi: 10.1186/1471-2164-9-67 (PMC2267717; doi:10.1186/1471-2164-9-67)
Supplement: Additional file 2 — Distribution of the 8-mer-association-with-RNAP for 8-mers containing particular dinucleotide. Histograms of the 8-mer-association-with-RNAP between -1,000 bp and +500 bp for abundant and all 8-mers with 8-mers containing each of the 10 dinucleotides noted in black. [file 1471-2164-9-67-S2.ppt]

## Slide 1
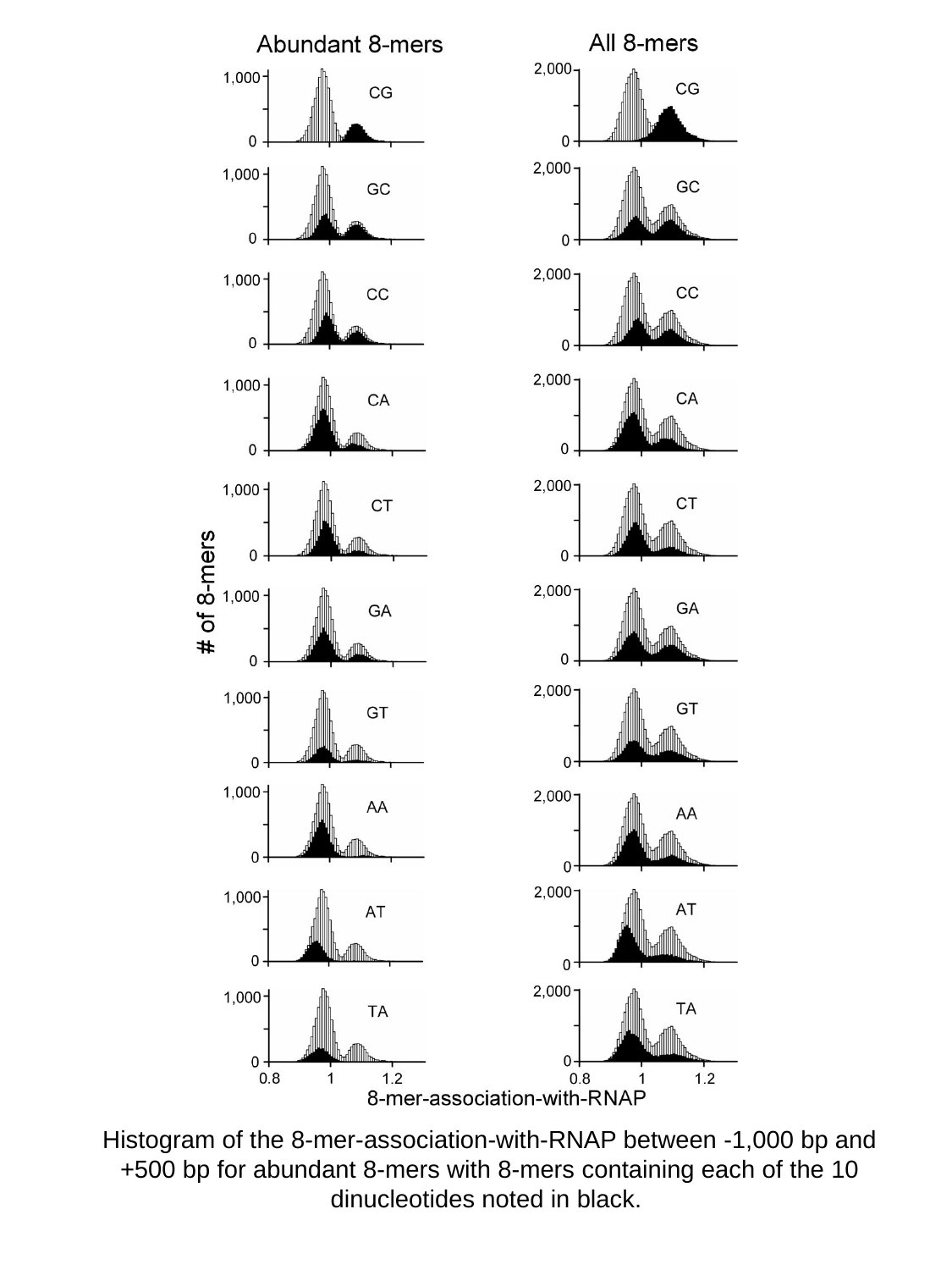

Histogram of the 8-mer-association-with-RNAP between -1,000 bp and +500 bp for abundant 8-mers with 8-mers containing each of the 10 dinucleotides noted in black.
