# Supplementary material for: All and only CpG containing sequences are enriched in promoters abundantly bound by RNA polymerase II in multiple tissues
Source: BMC Genomics. 2008 Feb 5;9:67. doi: 10.1186/1471-2164-9-67 (PMC2267717; doi:10.1186/1471-2164-9-67)
Supplement: Additional file 3 — Data presented at figures 2, 3(D–E), 5 and 6C for all 8-mers. The data presented at figures 2, 3(D–E), 5 and 6C is shown here for all 8-mers. Histograms and scatter plots for 8-mer-association-with-RNAP vs. 8-mer-association-with-H3K9me2, enrichment of 8-mers in 356 liver specific promoters vs. 8-mer-association-with-RNAP, clustering factor vs. 8-mer-association-with-RNAP. [file 1471-2164-9-67-S3.ppt]

## Slide 1
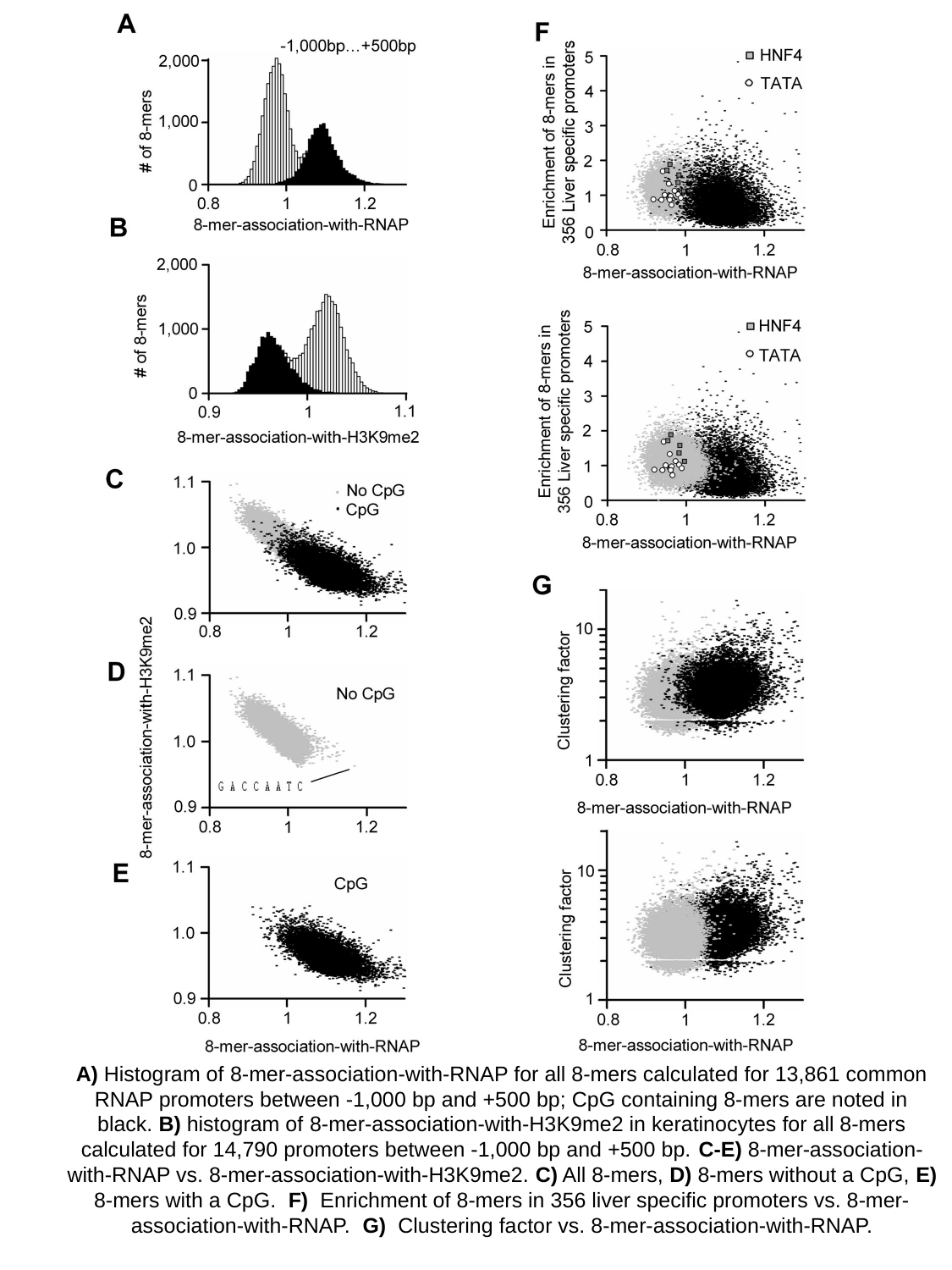

A) Histogram of 8-mer-association-with-RNAP for all 8-mers calculated for 13,861 common RNAP promoters between -1,000 bp and +500 bp; CpG containing 8-mers are noted in black. B) histogram of 8-mer-association-with-H3K9me2 in keratinocytes for all 8-mers calculated for 14,790 promoters between -1,000 bp and +500 bp. C-E) 8-mer-association-with-RNAP vs. 8-mer-association-with-H3K9me2. C) All 8-mers, D) 8-mers without a CpG, E) 8-mers with a CpG. F) Enrichment of 8-mers in 356 liver specific promoters vs. 8-mer-association-with-RNAP. G) Clustering factor vs. 8-mer-association-with-RNAP.
