# Supplementary material for: All and only CpG containing sequences are enriched in promoters abundantly bound by RNA polymerase II in multiple tissues
Source: BMC Genomics. 2008 Feb 5;9:67. doi: 10.1186/1471-2164-9-67 (PMC2267717; doi:10.1186/1471-2164-9-67)
Supplement: Additional file 4 — Supplementary tables. Table 1 shows the 120 statistically most non-randomly distributed sequences placed into 10 groups. Table 2 shows co-occurrence of the 10 proximal promoter motifs between -200 bp and the TSS in 14,790 mouse promoters, top 20% of common RNAP promoters and top 20% of promoters best bound by H3K9me2. [file 1471-2164-9-67-S4.ppt]

## Slide 1
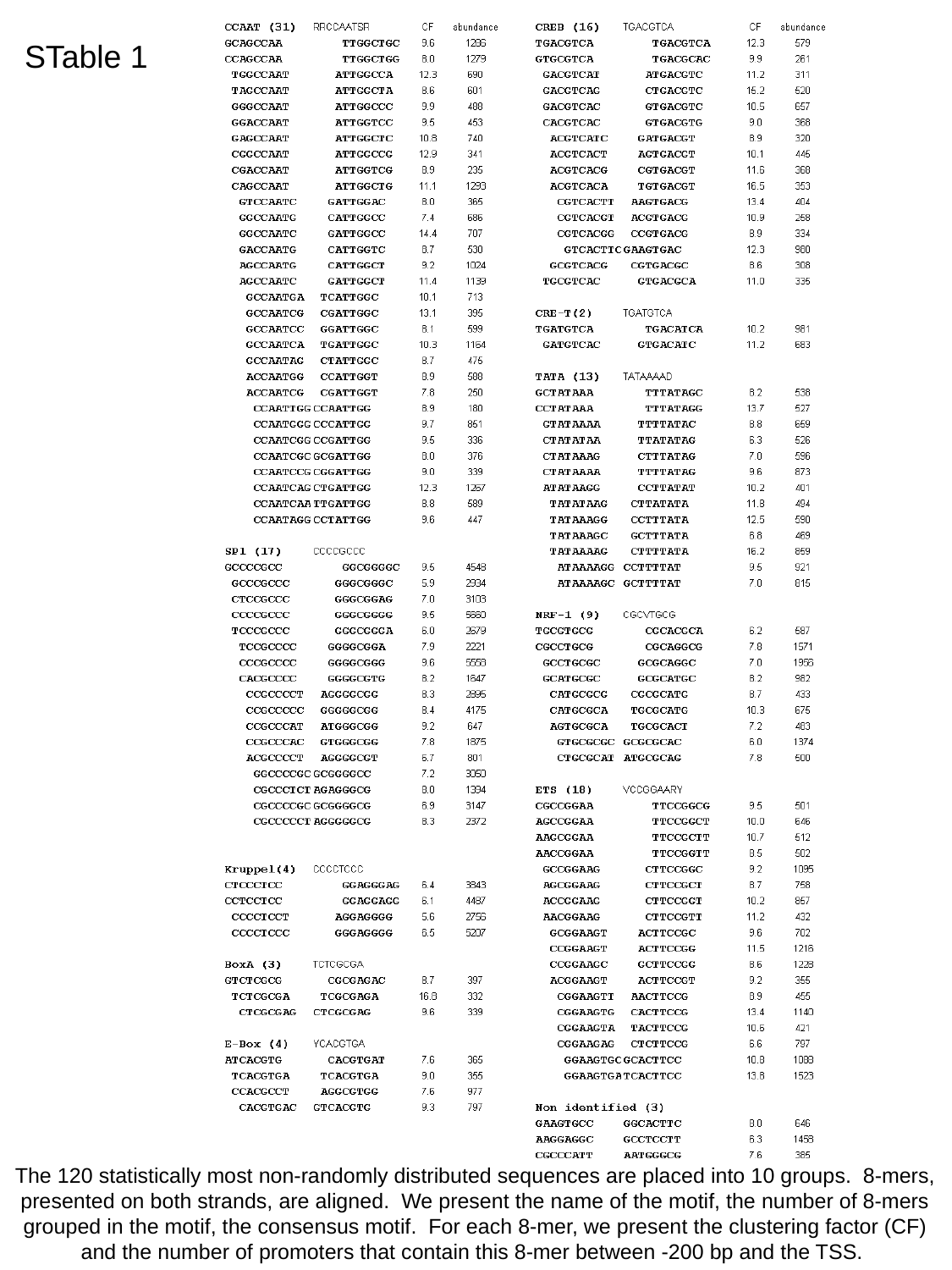

STable 1
The 120 statistically most non-randomly distributed sequences are placed into 10 groups. 8-mers, presented on both strands, are aligned. We present the name of the motif, the number of 8-mers grouped in the motif, the consensus motif. For each 8-mer, we present the clustering factor (CF) and the number of promoters that contain this 8-mer between -200 bp and the TSS.

## Slide 2
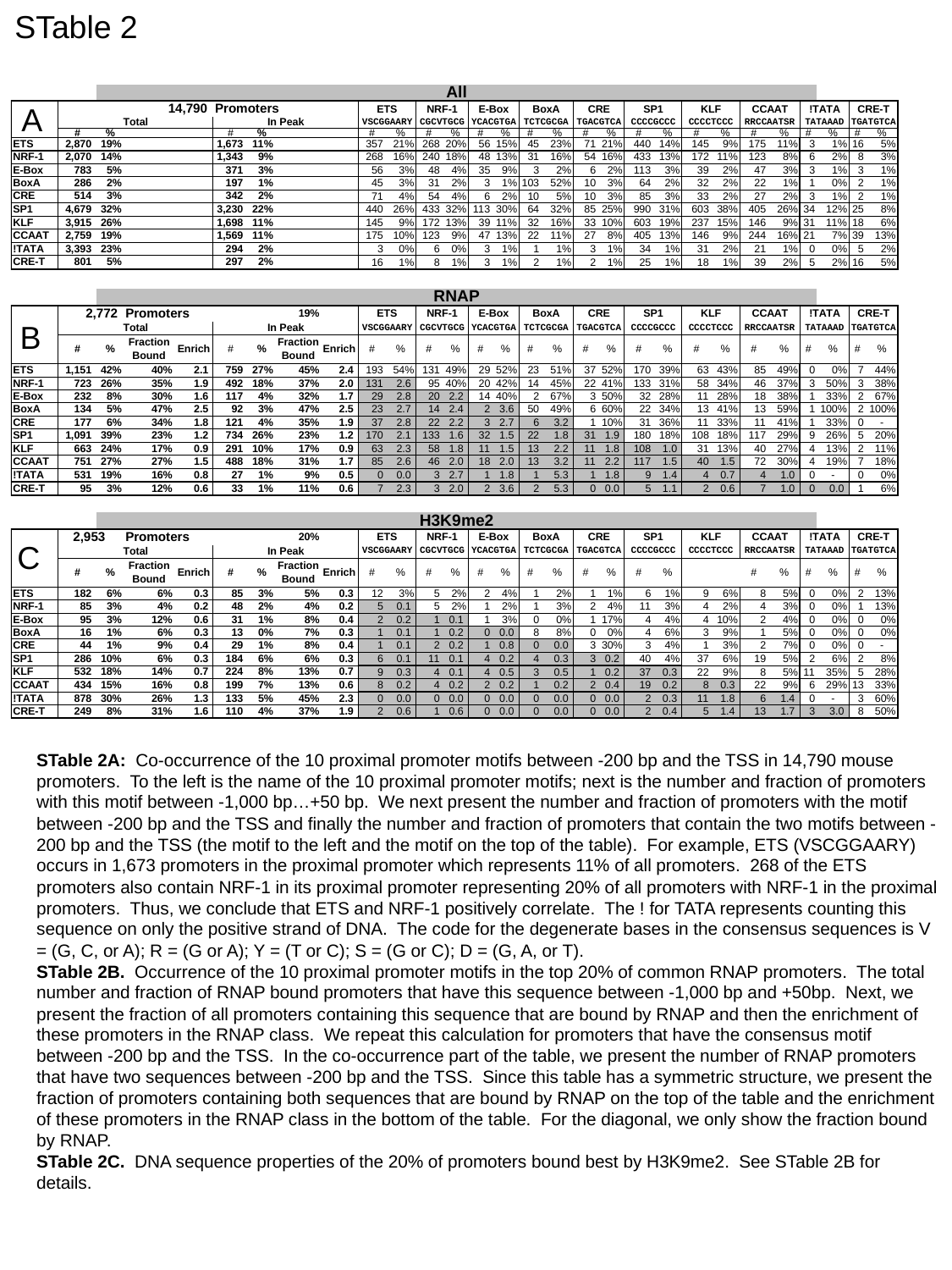

STable 2
A
B
C
STable 2A: Co-occurrence of the 10 proximal promoter motifs between -200 bp and the TSS in 14,790 mouse promoters. To the left is the name of the 10 proximal promoter motifs; next is the number and fraction of promoters with this motif between -1,000 bp…+50 bp. We next present the number and fraction of promoters with the motif between -200 bp and the TSS and finally the number and fraction of promoters that contain the two motifs between -200 bp and the TSS (the motif to the left and the motif on the top of the table). For example, ETS (VSCGGAARY) occurs in 1,673 promoters in the proximal promoter which represents 11% of all promoters. 268 of the ETS promoters also contain NRF-1 in its proximal promoter representing 20% of all promoters with NRF-1 in the proximal promoters. Thus, we conclude that ETS and NRF-1 positively correlate. The ! for TATA represents counting this sequence on only the positive strand of DNA. The code for the degenerate bases in the consensus sequences is V = (G, C, or A); R = (G or A); Y = (T or C); S = (G or C); D = (G, A, or T).
STable 2B. Occurrence of the 10 proximal promoter motifs in the top 20% of common RNAP promoters. The total number and fraction of RNAP bound promoters that have this sequence between -1,000 bp and +50bp. Next, we present the fraction of all promoters containing this sequence that are bound by RNAP and then the enrichment of these promoters in the RNAP class. We repeat this calculation for promoters that have the consensus motif between -200 bp and the TSS. In the co-occurrence part of the table, we present the number of RNAP promoters that have two sequences between -200 bp and the TSS. Since this table has a symmetric structure, we present the fraction of promoters containing both sequences that are bound by RNAP on the top of the table and the enrichment of these promoters in the RNAP class in the bottom of the table. For the diagonal, we only show the fraction bound by RNAP.
STable 2C. DNA sequence properties of the 20% of promoters bound best by H3K9me2. See STable 2B for details.
